# Practical Notes on Diagnosis and Treatment

**Published:** 1909-02-20

**Authors:** 


### PRACTICAL NOTES ON DIAGNOSIS AND TREATMENT.

### Consent to Operation.

A surgeon is justified in performing an operation upon a married woman with her consent if he deems it necessary for the prolongation of her life, and her husband has no right to withhold from his wife such surgical assistance as her case requires.—*Dr. S. B. Atkinson.*

### Lumbago.

A suitable application is menthol ointment—1 dram to ½ an ounce each of lanoline and vaseline. This can be thorougly applied, the fatty basis permitting continued friction. Acupuncture is another measure that often proves useful. The needles should be thrust deeply into the lumbar muscles at the site of the pain and allowed to remain for five to ten minutes.

### Distribution of Variolous Lesions.

The brunt of the attack falls upon the face and hands: the incidence on the face is greater than on any other part of the body. Of the upper limb, the lower limb, and trunk, the first is the most mobile and it sustains the thickest rash; the leg in most cases is less susceptible than the trunk, on the front of which the rash is more sparse than on any other part of the surface.—*Ricketts and Byles.*

### Change of Climate and Headache.

Where the headache has been induced by relaxing climatic conditions, a mixture containing two grains of quinine and five minims of liq. strychnin. will generally do all that is necessary. Where, on the other hand, the climate is "too strong," ten grains each of the iodide and bromide of potassium three times a day will be found useful. In each case the mixture should be preceded by a dose of calomel.—*Dr. Leonard Williams.*

### Carcinoma of the Prostate.

Carcinoma of the prostate is by no means a rare disease. It certainly occurs to the extent of ten per cent. in those cases of prostatic enlargement which are submitted to microscopical examination.—*Mr. Cuthbert Wallace.*

### Psoriasis.

The prognosis in psoriasis is favourable as far as any particular attack is concerned. Recurrence, however, after a longer or shorter period of complete or comparative freedom from the manifestations of the disease is the rule.—*Sir Malcolm Morris.*

### Neurasthenia.

A very useful combination for the treatment of the thin, constipated type of neurasthenic patient is as follows: Liquid extract of malt with glycerophosphates (B.P.), 7 oz.; liquid extract of cascara sagrada, 1 oz.; of which a tablespoonful may be ordered twice a day.

### Tuberculosis of the Kidneys.

I certainly have no doubt that tuberculosis in by far the greatest number of cases is primary in the kidneys, and I have been one of the first to maintain this view against Guyon's opposite opinion; but I consider it a serious error to suppose that tuberculosis cannot ascend.—*Professor Rovsing.*

### Compensation for Injury.

Twenty years ago nervous breakdown, *i.e.* an uninhibited nervous system, was scarcely recognised; at any rate it was too little recognised. Now we have gone to the other extreme. I am always chary of people who, when asked the nature of their injury, say "nervous shock to the system." They have generally been discussing the question of damages with a lawyer.—*Dr. R. J. Collie.*

